# Modified RTK-GNSS for Challenging Environments

**DOI:** 10.3390/s24092712

**Published:** 2024-04-24

**Authors:** Ellarizza Fredeluces, Tomohiro Ozeki, Nobuaki Kubo, Ahmed El-Mowafy

**Affiliations:** 1 Department of Marine Systems Engineering, Tokyo University of Marine Science and Technology, Tokyo 135-8533, Japan; etfredeluces1@gmail.com (E.F.); t1610111226@gmail.com (T.O.); 2 School of Earth and Planetary Sciences, Curtin University, Perth 6102, Australia; a.el-mowafy@curtin.edu.au

**Keywords:** RTK, navigation, multipath, reliability

## Abstract

Real-Time Kinematic Global Navigation Satellite System (RTK-GNSS) is currently the premier technique for achieving centimeter-level accuracy quickly and easily. However, the robustness of RTK-GNSS diminishes in challenging environments due to severe multipath effects and a limited number of available GNSS signals. This is a pressing issue, especially for GNSS users in the navigation industry. This paper proposes and evaluates several methodologies designed to overcome these issues by enhancing the availability and reliability of RTK-GNSS solutions in urban environments. Our novel approach involves the integration of conventional methods with a new technique that leverages surplus satellites—those not initially used for positioning—to more reliably detect incorrect fix solutions. We conducted three tests in densely built-up areas within the Tokyo region. The results demonstrate that our approach not only surpasses the fix rate of the latest commercial receivers and a popular open-source RTK-GNSS program but also improves positional reliability to levels comparable to or exceeding those of the aforementioned commercial technology.

## 1. Introduction

Real-time kinematic global navigation satellite system (RTK-GNSS) has been widely used in different positioning, navigation, and timing (PNT) applications that require high-accuracy results. It can provide up to centimeter-level accuracy by leveraging similar sets of measurements from at least two receivers, commonly known as base and rover stations, and use them to implement differencing techniques to eliminate most errors [1]. As for RTK-GNSS, carrier-phase measurements are used.

(1) $$\Phi_{k}^{p}\left(t\right)=\phi_{k}^{p}\left(t\right)-\phi^{p}\left(t\right)+N_{k}^{p}+S_{k}+f\tau_{k}-\beta_{iono}+\delta tropo$$

(2) $$\Phi_{m}^{p}\left(t\right)=\phi_{m}^{p}\left(t\right)-\phi^{p}\left(t\right)+N_{m}^{p}+S_{k}+f\tau_{k}-\beta_{iono}+\delta tropo$$

Equations (1) and (2), given by [1], represent the range of propagation paths between a satellite and two receivers based on carrier-phase measurements, where *k* and *m* are the receiver and receiver antenna phase centers, respectively; *p* is the satellite; $\phi^{p}$ is the transmitted signal phase as a function of time; $\phi_{k}^{p}\left(t\right)$ and $\phi_{m}^{p}\left(t\right)$ are the receiver-measured satellite signal phases as a function of time; *N* is the unknown integer number of carrier cycles from satellite *p* to *k* or satellite *p* to *m*; *S* is the phase noise due to receiver and multipath effects; *f* is the carrier frequency; τ is the associated satellite or receiver clock bias; $\beta_{iono}$ is the advance of the carrier (cycles) due to the ionosphere; and $\delta_{tropo}$ is the delay of the carrier (cycles) due to the troposphere. Differencing Equations (1) and (2) ($SD_{km}^{p}$), given by Equation (3), are calculated, in which signal phase and clock biases are eliminated. Tropospheric and ionospheric errors are also canceled, provided that the distance between receivers *m* and *k* is less than 50 km [1].

(3) $$SD_{km}^{p}=\phi_{km}^{p}+N_{km}^{p}+S_{km}^{p}+f\tau_{km}$$

If another satellite, say *q*, is considered and differencing is performed between receivers *m* and *k*, another $SD_{km}^{q}$ can be formed, as shown by Equation (4).

(4) $$SD_{km}^{q}=\phi_{km}^{q}+N_{km}^{q}+S_{km}^{q}+f\tau_{km}$$

Both $SD_{km}^{p}$ and $SD_{km}^{q}$ form combined integer ambiguity, a combined phase-noise value, and combined receiver-clock bias. Performing differencing again but this time with Equations (3) and (4), a new term, $DD_{km}^{pq}$, is formed, in which the remaining variables are the combined carrier-phase measurements at receivers *k* and *m* using satellites *p* and *q*, combined unknown integer ambiguities, and system phase noise due to receiver hardware biases and multipath effects [2].

(5) $$DD_{km}^{pq}=\phi_{km}^{pq}+N_{km}^{pq}+S_{km}^{pq}$$

By setting a reference satellite (in this, case satellite *p*), which is usually the satellite with the highest elevation, and repeatedly performing the same differencing as that expressed in Equations (1)–(4) but with all other visible satellites, a set of double-differenced measurements similar to Equation (5) will be formed. These will be primary input to different estimation methods used in RTK-GNSS, such as weighted least squares [3], Kalman filtering and its extensions [3,4], and factor graph optimization [5]. However, said estimation methods assume that all input parameters are float values and do not consider the integer nature of the double-difference integer ambiguity ($N_{km}^{pq}$). This issue is more commonly known as GNSS ambiguity resolution, which is conceptually divided into four steps, as described in [6]. The first step is to estimate the float parameters, disregarding the integer nature of ambiguity. The second step is further adjustment of real-valued ambiguities to take into account the integer constraints, which is usually performed by the LAMBDA method [7]. At this step, integer ambiguities are computed. The third step is verifying whether the computed integer ambiguities are to be accepted or not accepted as integer solutions [8]. Some common tests are the ratio test, distance test, and projector test, which are reviewed and evaluated in [9]. In the last step, when the integer solution has passed the validation test, the float solutions of all other parameters are corrected. It is called a "fixed solution", assuming that test in the third step is passed correctly.

Although RTK-GNSS can provide centimeter-level of accuracy, it is still a challenge to always obtain a relatively good positions in normal and dense urban areas. In fact, obtaining high-accuracy positions in challenging environments is of interest to many researchers and has its own session at the Institute of Navigation (ION), the biggest GNSS research conference as of now. Severe multipath effects and shortages of satellites with a direct line of sight (LOS) to the receivers due to the presence of many obstructions in urban areas largely contribute to the decrease in performance of RTK-GNSS. Hence, the improvement of RTK-GNSS in challenging environments strongly relies on whether satellites with non-line of sight (NLOS) or affected by multipath errors can be detected and excluded in the positioning [10]. Some research has addressed this by proposing the usage of 3D building models and fish-eye-view cameras to identify NLOS satellites and exclude them [11,12,13]. The integration of a GNSS/Inertial Navigation System (INS) also contributes by providing a continuous position, even in the event of GNSS signal outages, like in tunnels [14], and reducing large jumps due to multipath errors [15]. The advent of machine learning technology has also contributed by detecting NLOS and LOS satellites based on GNSS measurements [16] and GNSS signal characteristics [17].

In this study, the issue of achieving accurate positioning and reliable ambiguity resolution in challenging environments, such as dense urban areas, is addressed. Some traditional approaches are included, such as the use of GNSS velocity information and the resolution of integer ambiguity through the integration of various satellite systems. To enhance ambiguity resolution, both measurement and position domain approaches are employed, aiming to derive optimal outcomes. Furthermore, an effective satellite selection strategy incorporating a threshold value for the pseudo-range residual test is also explored.

The main contribution of this research is the improvement of reliability of RTK-GNSS solutions in challenging environments, such as urban and dense urban areas. Typically, these areas have compromised fixed positions due to multipath errors caused by tall buildings and other obstructions. Interestingly, a high availability of fixed positions can still be achieved in such areas. However, the issue arises when some of these fixed positions, when compared to reference fixed positions, exhibit large absolute errors. To improve reliability, an additional validation step for the integer solution is performed, which is applied after passing the ratio test. This novel approach contributes to the improvement of the reliability of RTK-GNSS, offering a more robust position in environments where conventional methods may be less effective.

The rest of this paper is organized as follows: Section 2 outlines and discusses the concept and implementation of improvements in RTK-GNSS in challenging environments. Section 3 presents the experiment performed around the dense urban area of Tokyo, Japan. Section 4 discusses the different RTK engines and parameter setups that will be used for comparison. Section 5 explains the results by comparing the proposed algorithm with existing commercial receivers and open-source RTK-GNSS libraries. Section 6 summarizes the findings and concludes the study.

## 2. Methodology

**Figure 1:**
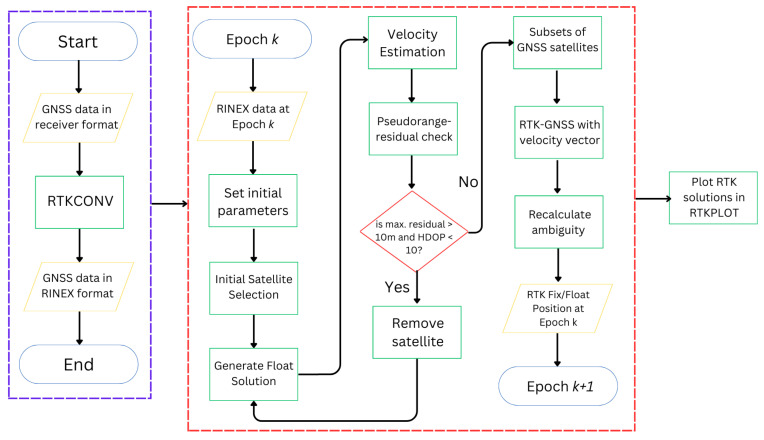
Implemented relative positioning method.

The source programs of RTKLIB [4] are the main tools used in this study, except that its relative positioning function, which is used for RTK-GNSS, is modified entirely to implement the proposed algorithms. These algorithms are henceforth referred to as Modified RTK-GNSS in this paper. The actual name of the function in RTKLIB that was modified is *relpos*. This section introduces the algorithms that are implemented in the *relpos* of RTKLIB. Figure 1 presents the general flow of Modified RTK-GNSS. Section 2.1 discusses Figure 1 in more detail, especially how satellite selection is performed. Section 2.2 focuses on the generation of a smoothed float solution. Section 2.3 explains the ambiguity resolutions using the velocity information obtained from Doppler frequency or time difference carrier-phase measurements. Section 2.4, which is the main contribution of this study, highlights how the integer ambiguity solution is further validated after it passes the ratio test.

### 2.1. Flowchart and Satellite Selection

GNSS raw measurements are converted from the receiver’s data format to RINEX format [18] using RTKCONV of RTKLIB, and the positioning solutions are visualized and analyzed using RTKPLOT of RTKLIB. The core part of the program is similar to RTKLIB, except that certain functions, such as satellite selection, the float solution, and additional checks of ambiguity by recalculating it, are modified and added to include the new concepts.

First, GNSS observation data from both the base and rover station were input. Thereafter, initial parameter settings and several satellite selection methods were performed. The pseudorange, Doppler frequency, and carrier-phase measurements had to be generated from the receiver. Furthermore, half-cycle ambiguities must be resolved by RTK-GNSS. The flag for this half-cycle checking from the receiver could be used for this purpose. The normal mask angle and required minimum $C/N_{0}$ are set for both base and rover stations. The mask angle is set to 10°, and the minimum $C/N_{0}$ is set to approximately 35 dB-Hz for the L1 band for urban environments as listed in Table 1. The minimum $C/N_{0}$ for the L2 band is also set because multi-frequency diversity can be expected [19]. The following signals are used for L1: GPS and QZSS L1 C/A, GLONASS G1, Galileo E1, and BeiDou B1I. The following signals are used for L2: GPS and QZSS L2C, GLONASS G2, Galileo E5b, and BeiDou B2. Because the wavelengths of L1 and L2 band frequencies of the same satellite system differ, the fluctuation period of $C/N_{0}$ between the two bands due to diffraction or reflection is different.

The minimum $C/N_{0}$ for L2P of GPS is not checked, since the commercial receiver that is used in this study does not output an L2P signal. In addition, the threshold of elevation-dependent $C/N_{0}$ is set. If the satellite is less than this threshold, then the satellite is not used [20]. After applying these settings as in the base station, the float solution of the rover is estimated by integrating the pseudorange-based DGNSS solution, Doppler-based velocity, and carrier phase, as will be discussed in Section 2.3. The residuals of the satellites after performing least-squares estimation are also checked. If a satellite has the maximum absolute residual and it is greater than 10 m, then said satellite is repeatedly removed from the set of satellites to use for positioning, provided that the HDOP is lower than 10. Finally, these measurements are used to obtain RTK-GNSS solutions, and the corresponding ambiguity is further validated when it has passed te ratio test. For the RTK-GNSS, several subsets of GNSS satellites are selected. For example, GNSS satellites with and without GLONASS are used. Here, the partial ambiguity resolution is also a better choice [21].

### 2.2. Generation of the Float Solutions

Equations (6)–(13) present a simple, basic system and estimation parameters for Kalman filtering, where *k* is the epoch count, *x_k_* is the state vector, *w_k_* is system noise, *y_k_* is the measurement vector, *v_k_* is measurement noise, *F* is the state transition matrix, *G* is the noise distribution matrix, and *H* is the observation matrix.

(6) $$x_{k+1}=Fx_{k}+Gw_{k}$$

(7) $$y_{k}=Hx_{k}+v_{k}$$

(8) $$x_{k}={\left[x\left(k\right),y\left(k\right),z\left(k\right),v_{x}\left(k\right),v_{y}\left(k\right),v_{z}\left(k\right)\right]}^{T}$$

(9) $$x\left(k+1\right)=x\left(k\right)+v_{x}\left(k\right)\Delta t$$

(10) $$y\left(k+1\right)=y\left(k\right)+v_{y}\left(k\right)\Delta t$$

(11) $$z\left(k+1\right)=z\left(k\right)+v_{z}\left(k\right)\Delta t$$

(12) $$F=\left[\begin{array}{cccccc} 1 & 0 & 0 & \Delta t & 0 & 0\\ 0 & 1 & 0 & 0 & \Delta t & 0\\ 0 & 0 & 1 & 0 & 0 & \Delta t \end{array}\right]$$

(13) $$H=\left[\begin{array}{ccc} 1 & 0 & 0\\ 0 & 1 & 0\\ 0 & 0 & 1 \end{array}\right]$$

Equations (14)–(19) show a portion of Kalman filtering that includes prediction, correction, and Kalman gain. Three-dimensional positions can be estimated using these equations. The inputs of this system are based on the ECEF coordinate system. Regarding the standard deviation as an input to the Kalman filter, the absolute position from the DGNSS is set as 2 m, and the velocity information is set as 2 cm/s.

(14) $${\hat{x}}_{k|k}={\hat{x}}_{k|k-1}+K_{k}\left(y_{k}-H_{k}{\hat{x}}_{k|k-1}\right)$$

(15) $$P_{k|k}=P_{k|k-1}-K_{k}H_{k}P_{k|k-1}$$

(16) $$K_{k}=P_{k|k-1}H_{k}^{T}{\left(I+H_{k}P_{k|k-1}H_{k}^{T}\right)}^{-1}$$

(17) $${\hat{x}}_{k+1|k}=F_{k}{\hat{x}}_{k|k}$$

(18) $$P_{k+1|k}=F_{k}P_{k|k}F_{k}^{T}+\left(\sigma_{w}^{2}/\sigma_{v}^{2}\right)\Lambda$$

(19) $$\Lambda =GG^{T}=diag\left\{0,0,0,1,1,1\right\}$$

**Figure 2:**
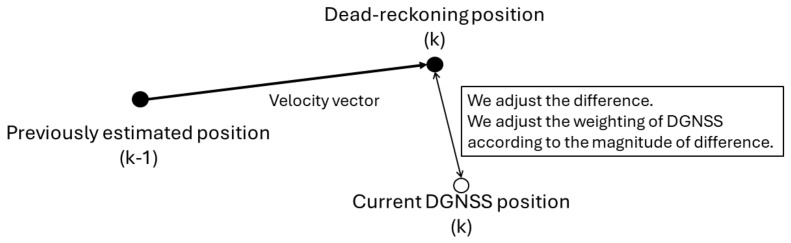
Outlier detection method.

Besides this algorithm, a simple dead reckoning using the previously estimated position by Kalman filtering and velocity vector is also implemented. To realize this, the calculated output ($x^{\prime }$, $y^{\prime }$, $z^{\prime }$) of dead reckoning is used. When the absolute difference between the dead-reckoning positions and the code-based DGNSS positions (*x*, *y*, *z*) is greater than 10 m, the DGNSS positions cannot be trusted because the pseudorange observations are highly deteriorated by a strong multipath effect. The weighting between (*x*, *y*, *z*) and ($x^{\prime }$, $y^{\prime }$, $z^{\prime }$) is adjusted according to the absolute difference. When the positions (*x*, *y*, *z*) are not trusted, the standard deviation of the Kalman filter is varied for the absolute position according to the magnitude of the estimated difference. A visualization of this method is shown in Figure 2.

This method is very effective in reducing the outliers obtained from the pseudorange-based DGNSS. The limitation of this method depends on the accuracy of velocity information. In that sense, satellite selection for velocity estimation is also very important. Even if the chosen satellite is valid in frequency-locked loop tracking, the velocity information deteriorates a lot in dense urban areas. In fact, the validation of the carrier-phase tracking should also be checked. Although they are not used in this paper, continuous measurement checks for the last several epochs will be effective for better satellite selection. With a prolonged outage of the GNSS of more than a few seconds due to the presence of enclosed areas such as overpasses and tunnels, it is important to reset the Kalman filtering appropriately.

### 2.3. Ambiguity Resolution Using Velocity Information

The LAMBDA method is used to search for correct ambiguities because this technique can search for the best solution using the integer least-squares method [7]. It is very important to resolve the ambiguities as quickly as possible to maintain reliability because there is a slight influence when cycle slips in the carrier phase occur in the phase-lock loop. A ratio test is used to determine if the ambiguities produced by the LAMBDA method are acceptable. The general threshold for the ratio test is set according to [22]. The RTK-GNSS used in this paper is supported by the Doppler frequency information to enhance the float solution, and its details are given in [23]. The Doppler-aided RTK-GNSS usually improves the fix rate by about 5 to 10%. The general HOLD mode of ambiguities in RTK is not used. HOLD means that the ambiguities are determined once and kept at the same values as long as those satellites do not have cycle slips. The GNSS constellations used in this study include GPS, GLONASS, Galileo, BeiDou, and QZSS. If four satellite systems (GPS + QZSS, GLONASS, Galileo, and BeiDou) are used with at least two satellites each, the minimum number of satellites is set as 8. In this case, 4 satellites are selected as primary satellites to generate double-difference observations. If GPS + QZSS, Galileo, and BeiDou are used with at least 2 satellites in each system for double differences, 3 satellites are selected as primary satellites in each satellite system, and the minimum number of satellites is 7. Ambiguity dilution of precision is also used for ambiguity resolution because it effectively decreases the number of incorrect positions [6]. The GPS L1C/A and L2C, QZSS L1C/A and L2C, GLONASS G1 and G2, Galileo E1 and E5B, and BeiDou B1 and B2 signals are checked. If the HDOP is above 10, the solution is not used.

When the “float solution” is calculated, double-differenced pseudoranges are typically used. If multi-epoch data can be used, not only pseudorange but also carrier-phase measurements can be used to smooth the pseudorange noise. However, multi-epoch data with sufficient carrier-phase measurements cannot be used, even for a short period. In general, the use of multi-epoch data is difficult for moving platforms in urban areas because of the frequent occurrence of signal blocking. Consequently, the probability of correct ambiguity fixing strongly depends on the pseudorange accuracy. If the pseudorange accuracy suffers from large multipath errors, it will be difficult to resolve the correct ambiguities, even if the LAMBDA method is used. Modified RTK-GNSS can mitigate this problem by taking advantage of user velocity information.

**Figure 3:**
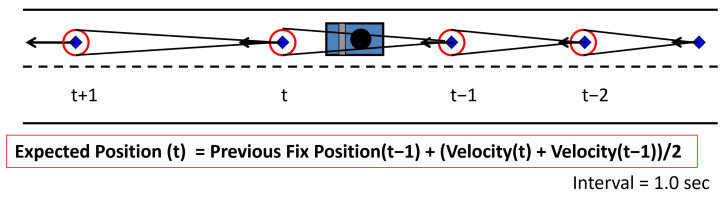
Concept of ambiguity resolution using velocity information.

Specifically, the “new float solution” is calculated by updating an accurate previous position with velocity information. An accurate previous position is critical here. To decide if the previous epoch was correctly fixed or not, the ratio test and recalculated ambiguity are checked, as will be discussed in Section 2.4. If the previous epoch passed the ratio test and the recalculated ambiguity test, the previous position is regarded as the result of correct ambiguities. Once the correct ambiguities are resolved, the “float solution” in the next epoch will be accurate and reliable. This is very important to strengthen the mathematical model because the LAMBDA method itself is not strong against bias errors. As for the resolution of the correct ambiguity, there are more effective methods, such as using multi-epoch measurements. The method to involving the use of the velocity at each epoch is described as follows. For a moving platform such as a car, a certain degree of acceleration has to be considered. Thus, the expected position is the previous fixed position updated by adding half of the present velocity estimate and half of the previous velocity estimate. Figure 3 shows this method. Even if ambiguities cannot be fixed and a fixed position cannot be obtained at the current epoch, the position accumulated by the velocity vector will be used for a few seconds because the accuracy of these positions is better relative to that of pseudorange-based DGNSS positions in urban environments.

### 2.4. Additional Validation of Ambiguity after Ratio Test

The ratio test is not always sufficient to validate the ambiguity after using LAMBDA as the fixed threshold, which is just based on empirical values and has its shortcomings [24].

**Figure 4:**
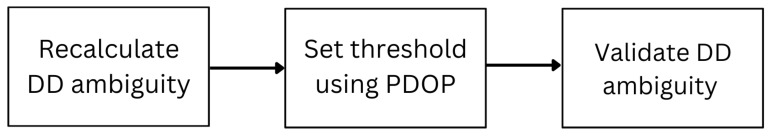
General flow of validating the ambiguity solution after passing the ratio test.

To address this issue, a further validation test is performed on the recalculated double-differenced (DD) ambiguity based on the supposed fixed position that passed the ratio test. The general steps are shown in Figure 4 and are discussed in more detail in this section.

#### 2.4.1. Recalculating Ambiguity

If the integer ambiguity solution passes the ratio test (i.e., the ratio of first and second ambiguity candidates is greater than the set threshold), the fixed position is used to recalculate the DD ambiguity of satellites in which the reference satellite, like *p* in Equation (5), is intentionally not included in RTK-GNSS processing. That way, the check will be independent from the double-differencing results in RTK-GNSS. Visible GPS Block IIR satellites and QZSS satellites with the highest elevation angle at the current epoch are selected as set of reference satellites for double differencing and consequently excluded in RTK-GNSS processing. All GPS Block IIR satellites output only L1C/A observables.

In the RTK-engine that is used, these satellites are automatically excluded because of the setting requiring the use of only satellites with both L1C/A and L2C observables. Although QZSS satellites have both signals, the highest QZSS satellite at the current epoch is also selected and excluded in RTK-GNSS to provide an additional independent check, since not all GPS Block IIR satellites are visible to the receiver, especially in urban environments. Recalculation of DD ambiguity is only performed for GPS/QZSS satellites and L1C/A observables, but it can be extended to other satellite constellations and other frequencies as long as they will be intentionally excluded for calculation of the fixed position in RTK-GNSS, just like for the QZSS with the highest elevation.

(20) $$N_{km}^{pq}=\phi_{km}^{pq}-DD_{km}^{pq}+S_{km}^{pq}$$

Derived from Equation (5), DD ambiguity can be calculated using the supposed fixed position that passed the ratio test to obtain the geometric range between a receiver and a satellite. Theoretically, if the position is correctly fixed, the recalculated ambiguity should be close to an integer value. For example, if the recalculated DD ambiguity is 4.95 and its nearest integer value is 5, there will be an absolute difference of 0.05. This absolute difference should be as small as possible to mathematically say that the RTK position is correctly fixed.

#### 2.4.2. Setting Up the Threshold

**Figure 5:**
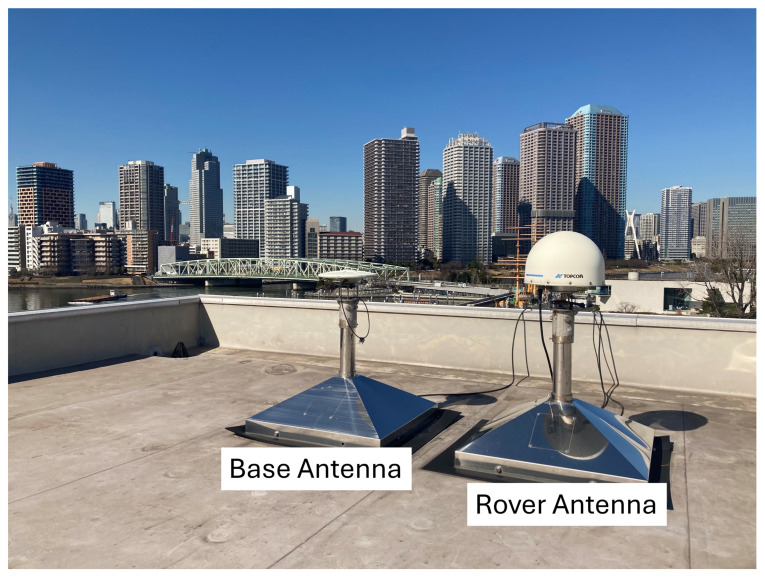
Rover and base antenna on the rooftop for static test.

**Figure 6:**
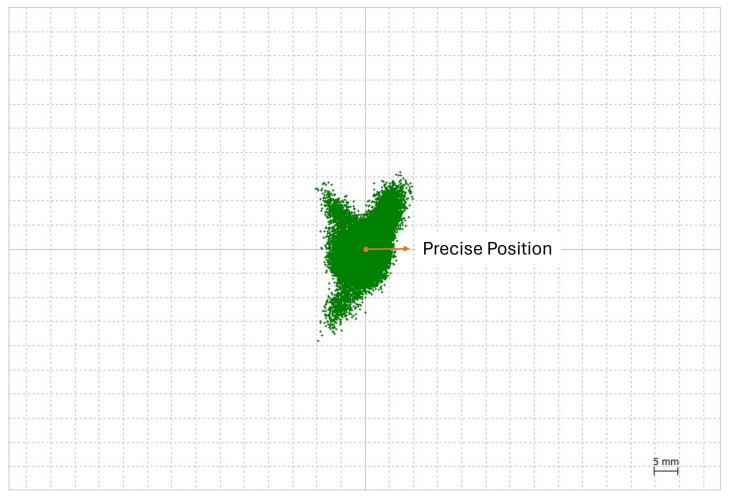
RTK position results of antenna located on rooftop.

**Figure 7:**
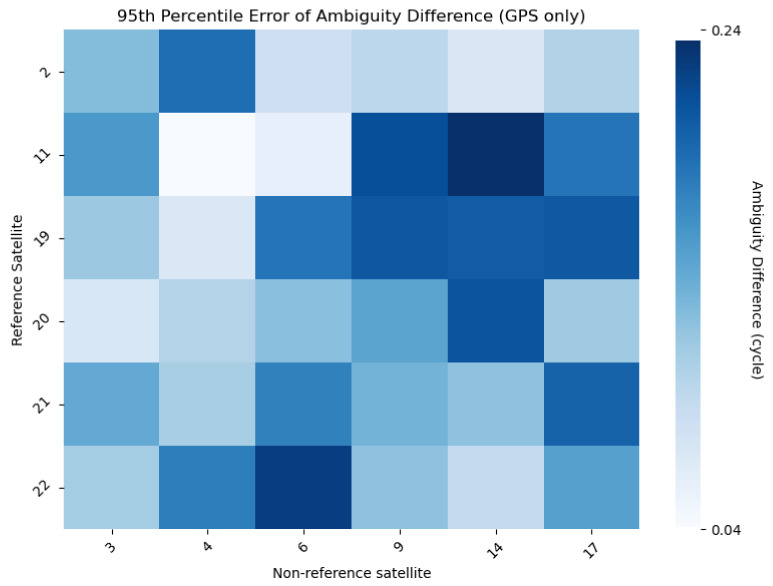
Summary of 95th percentile error of ambiguity difference for every double-difference pair.

To set a basis for a threshold, RTK processing is performed with a static dataset in an open-sky environment. The antennas of rovers and bases are located on the rooftop of a building in TUMSAT, which has a baseline length of around 1 m, as shown by Figure 5. The average position of the RTK processing results is set as the precise position of the rover antenna. Figure 6 shows the plot of RTK processing results and the precise position, which are represented by green marks and an orange mark, respectively. These results have a 95th percentile error of 1 cm in the horizontal direction and 2 cm in the vertical direction. Using the precise position and 95th percentile errors, DD ambiguities are recalculated for this dataset, where visible GPS Block IIR satellites are used as reference satellites. As shown in Figure 7, the differences between the recalculated DD ambiguities and the corresponding nearest integer are calculated; this will be the ambiguity difference. The 95th percentile errors of ambiguity difference are calculated for every pair and are summarized in Figure 7; these values range from 0.04 to 0.24 cycle. This shows that even in an open-sky environment, where GNSS signals are within the direct LOS of the receiver, which means that the carrier phases are expected to be of the best quality, the ambiguity difference is already around 0.2 cycles, even after just introducing a 1 to 2 cm error in the position. In urban environments, the introduced error will be higher than the previous ones. As checked with our datasets, 95th percentile errors of RTK position results of datasets collected in urban environments are 4 to 6 cm. By introducing these errors in the same empirical test, ambiguity differences are expected to be larger in dense urban areas than in open-sky environments due to multipath errors causing the degradation of GNSS signal quality.

A threshold based on position dilution of precision (PDOP) is used. PDOP is used because of its relationship to position accuracy. Improvement in PDOP, which means better satellite geometry, contributes to improvement of position accuracy [25,26,27]. In dense urban areas, PDOP is degraded because of obstructions present in such areas, while it is lower in open-sky environments.

Table 2 shows a summary of the thresholds that were used based on the PDOP value. Essentially, a smaller threshold should be used in open-sky environments because the PDOP value is better than in urban or dense urban areas because of good satellite visibility. The likelihood of multipath error affecting the position result in open-sky environments is smallest or can be nonexistent because of the higher visibility of LOS satellites. On the other hand, a larger threshold should be used in dense urban areas because it is expected that multipath errors will have greater effects due to presence of many obstructions. As shown in the empirical test, around 0.2 cycles is the maximum range of ambiguity difference in open-sky areas, but the most frequent is 0.1. For this reason, 0.1 is used for best PDOP values, which are determined to be less than 1 in this case. To obtain the general case, PDOP values between 1 and 2 (inclusive) have a threshold of 0.2. At this point, the threshold setting is still based on an empirical test done. For the worst PDOP, which is expected to occur in dense urban areas, PDOP values greater than 2 have a threshold of 0.3.

#### 2.4.3. Validate Recalculated DD Ambiguity

**Figure 8:**
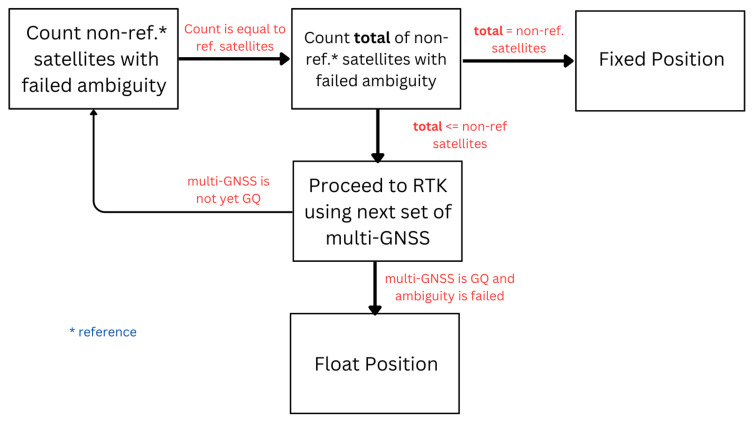
Validating recalculated DD ambiguity.

Figure 8 illustrates the method of validating recalculated DD ambiguity. The recalculated DD ambiguity is compared to the threshold based on GDOP. If it is greater than the threshold, then it is marked as failed. Otherwise, it is marked as passed. At every epoch, this is performed iteratively on every satellite pair for DD where the reference satellite is changed. Therefore, if we have 4 visible reference satellites to check, there will be 4 iterations for each non-reference satellite where we check the recalculated ambiguity. If all of non-reference satellites has failed ambiguity for each reference satellites, then the integer ambiguity solution calculated by LAMBDA, verified by ratio test, and used to compute the fixed position cannot be trusted. Hence, the supposed fixed position will be rejected and declared a float solution.

The iterative check described above is performed utilizing the advantage of the multi-GNSS constellation. First, the supposed fixed position is determined using GPS, QZSS, Galileo, Beidou, and GLONASS (GQEBR). If the check fails even if the ratio test is passed, another supposed fixed position is calculated but with only GPS, QZSS, Galileo, and Beidou (GQEB) and so on. The order is GQEBR, GQEB, GQER, GQE, GQB, and GQ. This is repeated until the ratio test and ambiguity check are not passed and there are still enough satellites. When the ambiguity check still fails after utilizing multi-GNSS, the position is declared a float solution.

## 3. Data Collection

**Figure 9:**
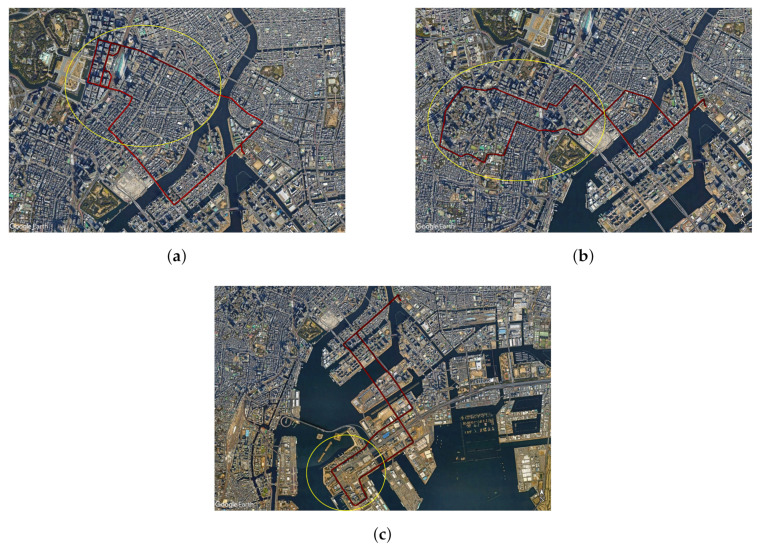
Experimental test courses. (**a**) First test course in Marunouchi and Ginza areas. (**b**) Second test course in Toranomon and Shimbashi areas. (**c**) Third test course in Odaiba area.

A car experiment was performed in three different test courses around Tokyo, Japan. Figure 9 shows these test courses, where the red marks are the ground track points and the yellow circles highlights the urban areas.The first test course is around Marunuochi and Ginza areas, which include many high-rise buildings. The second test course is around Toranomon and Shimbashi, where many high-rise buildings and overpasses are present. The third test course is in Odaiba, including some medium-rise buildings and overpasses. There are some NLOS environments in both the first and second test courses. There are pedestrian bridges and river bridges in all test courses. For all test courses, the start and finish points are located in TUMSAT.

**Figure 10:**
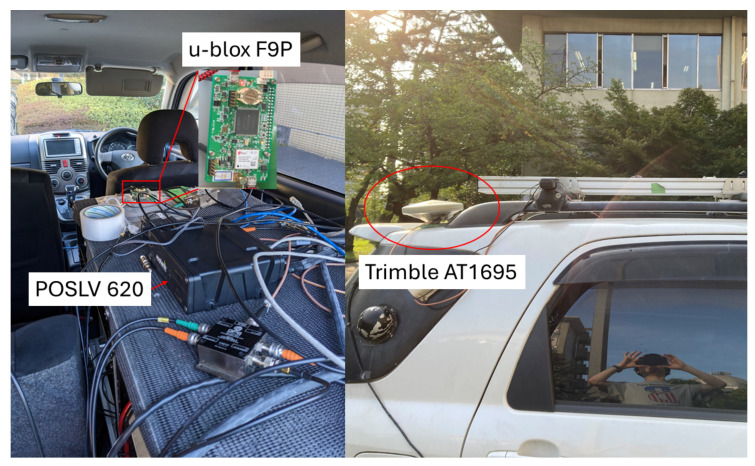
Setup of car experiment.

**Figure 11:**
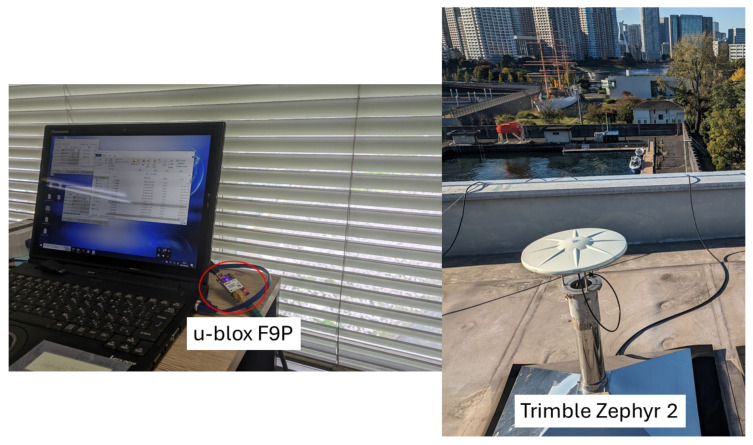
Setup of base station.

The setup of the experiment is shown in Figure 10. Two receivers connected to the same antenna via a GNSS splitter, were mounted in the car to collect data during the experiment. One is a u-blox F9P [28], and the other is a POSLV 620 [29]. Raw data (e.g., pseudorange, carrier-phase, and Doppler-frequency observables) and RTK positions of u-blox F9P were logged. To obtain RTK positions during the experiment, it is connected via NTRIP to a base station located at the TUMSAT Etchujima Campus. The receiver and antenna used in the base station are shown in Figure 11, which are u-blox F9P and Trimble Zephyr 2 [30], respectively. Simultaneously, reference RTK positions were collected by POSLV 620 which will be used for comparison of Modified RTK-GNSS with other RTK engines. The sampling rate of the raw data and RTK positions was 5 Hz. The total data recording periods for three different courses were 3360 s (16,800 epochs in 5 Hz), 3088 s (15,440 epochs in 5 Hz), and 2852 s (14,260 epochs in 5 Hz), while the sampling rate of base station data was 1 Hz.

## 4. Obtaining Fixed Solutions from Different RTK-Engines

The raw data of the GNSS were post-processed using the Modified RTK-GNSS mentioned earlier. The base station on the rooftop of Number 4 Building of the TUMSAT Etchujima Campus was used for data correction. The baseline length was less than 10 km in each test. Settings of the important parameters were kept the same for all tests, as follows: mask angle was set to 10 degrees, the minimum $C/N_{0}$ was set to 35 dB-Hz, the threshold for pseudorange residual check was set to 10 m, and the code/carrier ratio was set to 100.

The fixed positions calculated using the Modified RTK-GNSS were compared and evaluated against three different RTK-engines, namely u-blox F9P, RTKLIB, and rtklibexplorer [31]. The processing was solely conducted in forward mode. For all RTK-engines, the same base station was utilized for both post-processing and real-time data correction.

The fix position results for the u-blox F9P were computed by the receiver itself when connected to the base station via NTRIP, with the output provided in NMEA format. For RTKLIB and rtklibexplorer, two sets of parameters were employed. First, the same parameters, such as mask angle, $C/N_{0}$ mask, and carrier/code ratio, were used across both programs and were identical to parameters used in the Modified RTK-GNSS. Second, the presumed optimal parameters for RTKLIB and rtklibexplorer were used and varied in each test course.

To determine the optimal parameters for the two libraries, the parameter settings were iteratively checked to find the best performance. The parameter settings included minimum $C/N_{0}$ ratio, mask angle, code/carrier-phase error ratio, and the minimum ratio required to fix ambiguities. For both programs, GLONASS satellites were not used because they did not contribute anything to fix rate and accuracy. The reason behind this needs to be investigated further and is not part of the scope of this paper. The use of dual-frequency observables was set, and only the forward mode was used. The minimum threshold for the ratio test was set to 3.0. If the threshold were set lower than 3.0, such as 2.5 or 2.0, the fix rate would increase, but the accuracy would be deteriorated.

Table 3 summarizes the setting values of optimal parameters for RTKLIB. As for the ambiguity resolution method, the instantaneous mode was used because it performed best in te three modes using RTKLIB in urban areas. For the first test course, the optimal settings were a mask angle of 10 degrees, a minimum of 40 dB-Hz, and a code/carrier ratio of 200. The same settings were identified as the best for the second test course. For the third test course, the optimal settings were a mask angle of 30 degrees, a minimum of 40 dB-Hz, and a code/carrier ratio of 100.

Table 4 summarizes the setting values of parameters for rtklibexplorer. As for the ambiguity resolution method, the fix-and-hold mode is used because it achieves the best results in the three modes using rtklibexplorer in urban areas. For the first test course, the optimal settings were identified as a mask angle of 30 degrees, a minimum of 42 dB-Hz, and a code/carrier ratio of 300 (minimum lock time to fix ambiguity = 10). For the second test course, the best settings were a mask angle of 30 degrees, a minimum of 40 dB-Hz, and a code/carrier ratio of 300 (minimum lock time to fix ambiguity = 5). For the third test course, the optimal settings were a mask angle of 25 degrees, a minimum of 42 dB-Hz, and a code/carrier ratio of 200 (minimum lock time to fix ambiguity = 10).

## 5. Results

The evaluation and discussion of results will be based on fix rate and 2DRMS values of position results that include urban environments. This section includes the following: (1) a comparison of the float solutions of Modified RTK-GNSS and u-blox F9P, (2) comparison of fixed solutions with and without using the Modified RTK-GNSS, and (3) comparison of fixed solutions of the Modified RTK-GNSS with different RTK engines.

### 5.1. Comparison of Float Solutions

**Figure 12:**
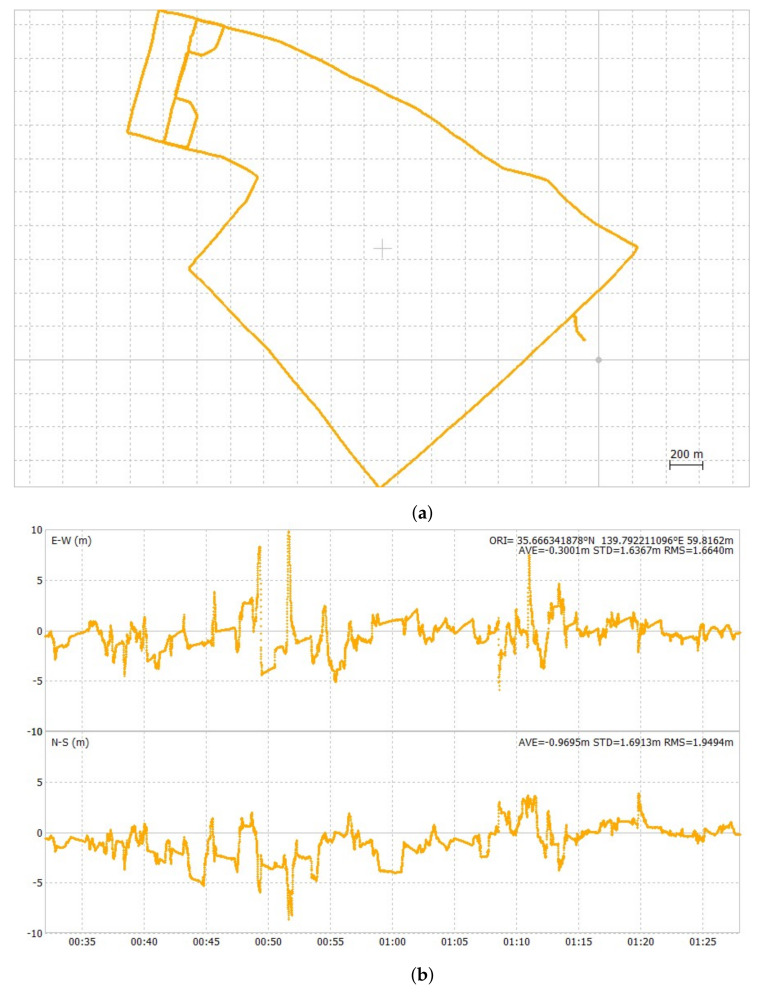
Ground track and horizontal time-series error plot of float solutions of first test course.

**Figure 13:**
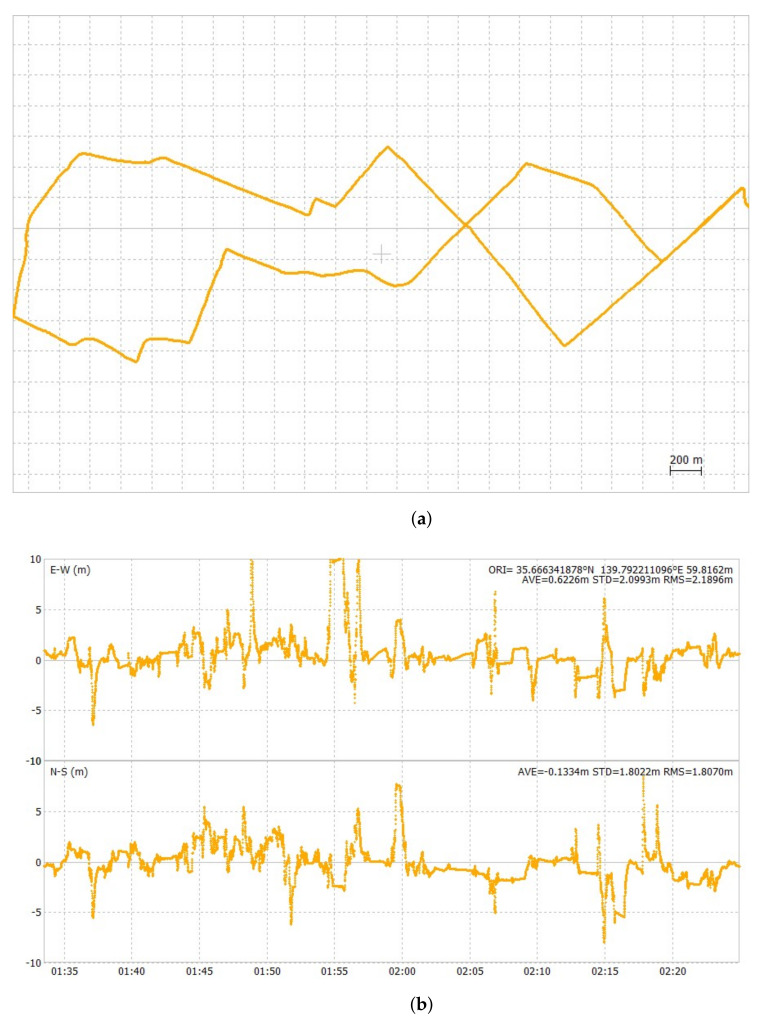
Groundtrack and horizontal time-series error plot of float solutions of second test course.

**Figure 14:**
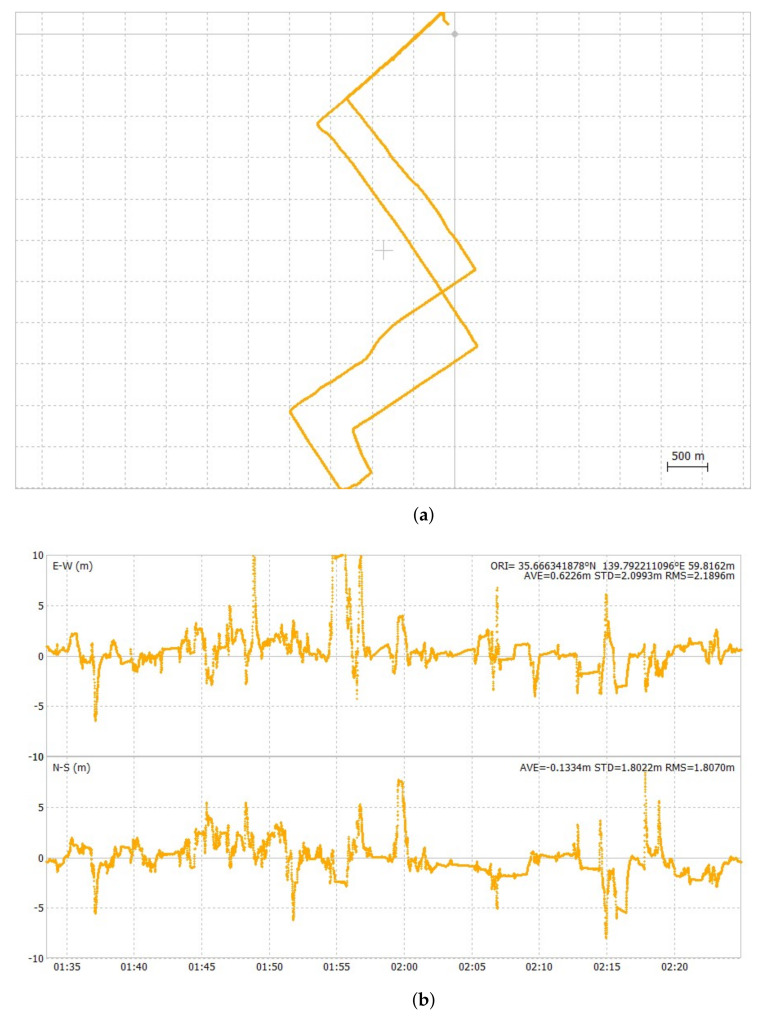
Groundtrack and horizontal time-series error plot of float solutions of third test course.

Figure 12, Figure 13 and Figure 14 show the ground track of float solutions and its time-series error plots for each test course. Only float solutions are shown here for all test courses to evaluate the pure float solutions. Figure 12a,b are the float solution results of the first test course. Its 2DRMS value of horizontal errors was 5.12 m. Figure 13a,b show the float solution results of the second test course. Its 2DRMS value of horizontal errors was 5.68 m. Figure 14a,b show the float solution results of the third test course. Its 2DRMS value of horizontal errors was 8.41 m. The center of the horizontal plots is set as the average of all the positions. The temporal errors were determined by obtaining the difference between the results of POSLV 620 and the results of the float solutions. The third test course has the highest 2DRMS value among all three test courses, which can be attributed to the presence of several train overpasses. At this location, half of the sky view was masked, and float solutions cannot cope well with such conditions. Table 5 summarizes the accuracy of float solutions compared with the test results of u-blox F9P, which were collected simultaneously. The results of u-blox F9P include all solutions, such as RTK-GNSS, float solutions, and DGNSS. On the other hand, the test results of the Modified RTK-GNSS are the integrated solutions mentioned in Section 2.3. It does not include RTK-GNSS. At this point, the float solutions of the Modified RTK-GNSS and u-blox F9P cannot be compared directly because u-blox F9P does not output the raw float solution results.

### 5.2. Comparison with Other RTK Engines

This section evaluates the fixed solutions of the Modified RTK-GNSS against u-blox F9P, RTKLIB, and rtklibexplorer, which are presumed to not implement the same further ambiguity validation that was performed in this study.

#### 5.2.1. Comparison with u-blox F9P

**Figure 15:**
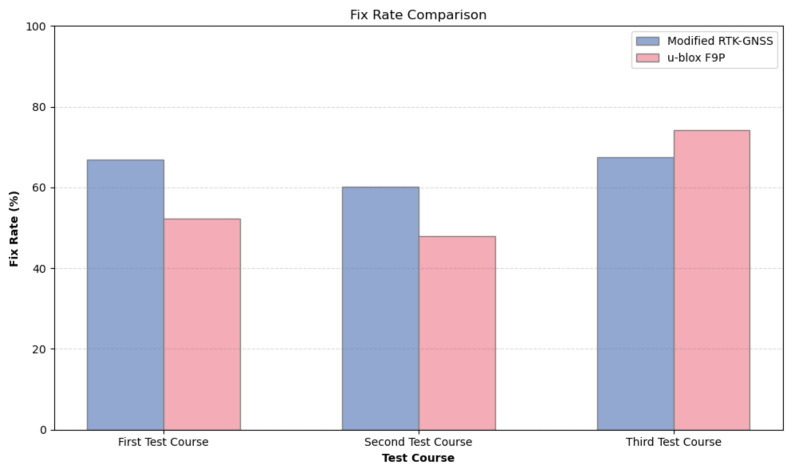
Fix rate comparison of Modified RTK-GNSS and u-blox F9P.

Figure 15 shows a visual comparison of the fix rate, while Table 6 details the 2DRMS values, offering insights about the reliability of the output solution for each RTK engine. Modified RTK-GNSS shows a higher fix rate coupled with a better 2DRMS value in the first test course and slightly worse values in the second test than u-blox F9P. This suggests that the Modified RTK-GNSS has good availability and reliability of the fixed position in the type of urban environment of the first and second test courses. In the second test course, some wrong fixes with very large errors are still classified as fixed position, which contributes to the calculation of 2DRMS. However, in the third test course, although the Modified RTK-GNSS shows a lower fix rate (approximately 6.7% less than that of u-blox F9P), it maintains a better 2DRMS value. This indicates that while the accuracy (reliability) of the the proposed method is upheld, the consistency in achieving fixed positions (availability) is compromised.

**Figure 16:**
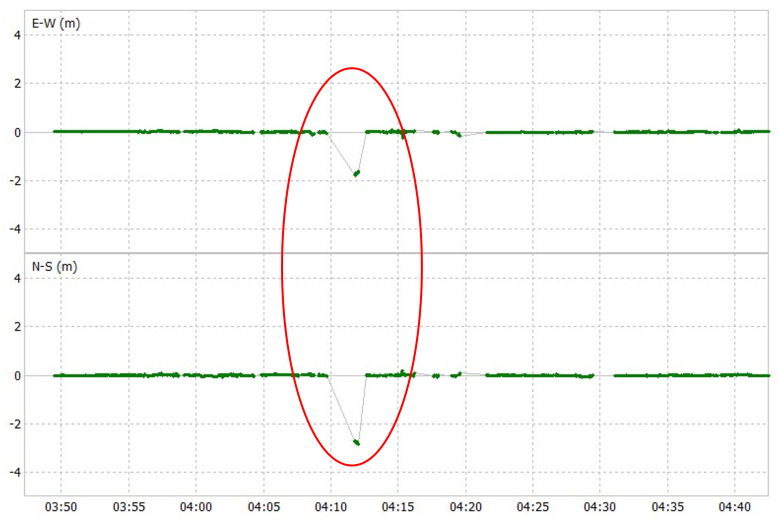
Horizontal plot of u-blox F9P showing large errors (encircled region).

**Figure 17:**
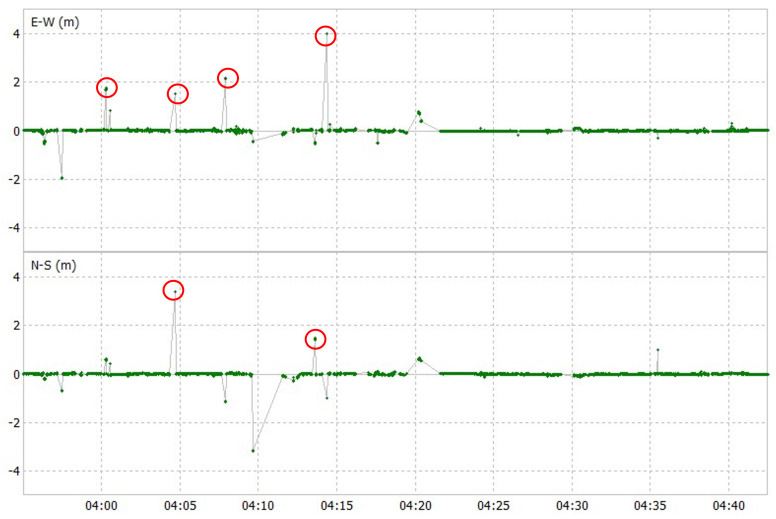
Horizontal plot of Modified RTK-GNSS showing large errors (encircled region).

There are certain consecutive epochs where u-blox F9P has large errors when compared to POSLV 620. An example is shown in Figure 16, which is a horizontal error plot of u-blox F9P in the third test course. Although only certain parts have large errors, this lasted for a few epochs, degrading its 2DRMS. Although some large errors of fixed position can also be seen in Modified RTK-GNSS and partly scattered in some epochs, such occurrences are fewer. The third test course is shown as an example in Figure 17. This shows that obtaining a higher fix rate will not always lead to better accuracy, especially in urban environments where multipath error is prominent.

#### 5.2.2. Comparison with RTKLIB and Rtklibexplorer

The use of the same parameters across all three RTK-GNSS programs is initially discussed. The primary aim is to assess the effectiveness and robustness of the Modified RTK-GNSS in an urban environment compared to the other two RTK engines. Table 7 summarizes the fix rate of the Modified RTK-GNSS, RTKLIB, and rtklibexplorer. In the first test course, Modified RTK-GNSS significantly outperformed both programs by approximately 22% to 42%. In the second test course, the fix rates of Modified RTK-GNSS and rtklibexplorer exhibited a 10% difference, while the difference between Modified RTK-GNSS and RTKLIB was 35%. In the third test course, the difference in fix rate between Modified RTK-GNSS and rtklibexplorer narrowed to 1%, and the difference relative to RTKLIB was 26%. This means that in terms of availability of fix positions, the Modified RTK-GNSS is superior in all three test courses.

**Figure 18:**
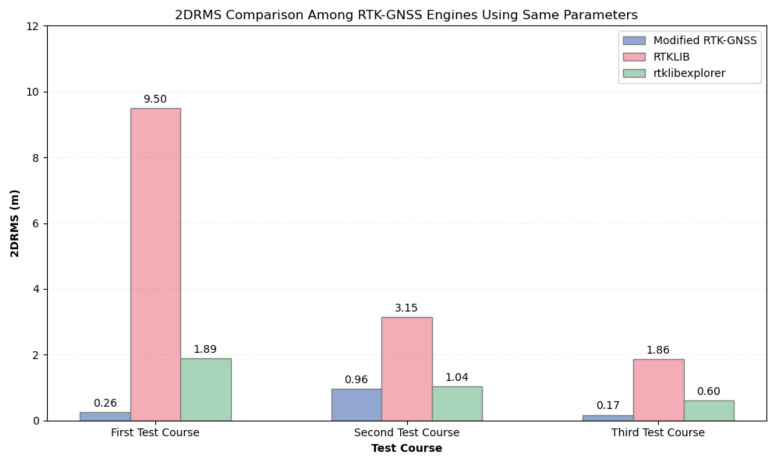
2DRMS comparison of three RTK-GNSS engines using **the same parameters**.

Figure 18 visually presents the 2DRMS values across three RTK engines, where same parameters were used. Modified RTK-GNSS clearly outperformed both open-source libraries by significant margins in this comparison. Since we use the same basic parameters in all three programs, these results can be attributed to the improvement of RTK-GNSS with ambiguity, which enables it to output more correct fixes and fewer wrong fixes.

**Figure 19:**
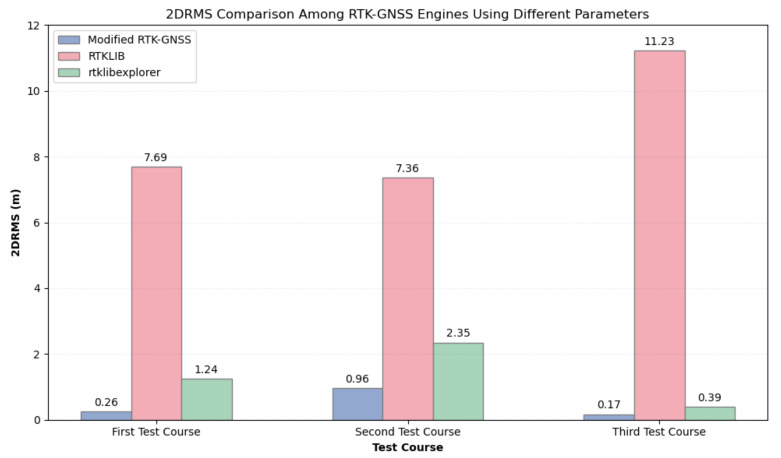
2DRMS comparison of three RTK-GNSS engines using **different parameters**.

Table 8 and Figure 19 show the fix rate comparison and 2DRMS values, respectively, of three RTK engines when the optimal or best parameters were used in RTKLIB and rtklibexplorer. It can be noticed that rtklibexplorer has the highest fix rate values in all test courses, but the reliability of fix positions in terms of 2DRMS is compromised. The fix-and-hold ambiguity mode helped rtklibexplorer to increase the fix rate, but it also degraded the output because the integer ambiguity might be erroneous. This means that obtaining high fix rates will not automatically lead to good accuracy of fix positions because of the risk of obtaining more wrong fixes, too. The Modified RTK-GNSS addresses this issue. Although it has a lower fix rate than rtklibexplorer’s results, Modified RTK-GNSS was able to capture more correct fixes and reject more wrong fixes than rtklibexplorer, as reflected by its better 2DRMS values in all test courses. RTKLIB has the lowest fix rate and largest 2DRMS values in this comparison, primarily because this library has yet to be improved by incorporating algorithms that enhance RTK position in urban environments.

In both comparisons, some improvement can be seen in 2DRMS values of RTKLIB and rtklibexplorer when they use the same parameters as the Modified RTK-GNSS. This is because the availability of fix positions decreased, and most probably wrong fixes are also reduced. However, the good balance of availability and reliability should be maintained, as is addressed by the Modified RTK-GNSS.

## 6. Conclusions

This study proposed a combination of conventional methods for the enhancement of satellite selection used for RTK-GNSS positioning and Doppler-based velocity information to improve ambiguity resolution. An additional validation of the integer solution after the ratio test was also performed with the aim of improving the reliability of fixed position results. The results of the Modified RTK-GNSS were compared against popular RTK-GNSS programs. One of those is the u-blox F9P receiver, which has built-in RTK engine processing in its chip and works for real-time processing. The other two are RTKLIB and rtklibexplorer, which is a forked repository of the former. The points of comparison were fix rate and 2DRMS. Compared against u-blox F9P, Modified RTK-GNSS had a superior fix rate and 2DRMS values in the first and second test courses, which suggests the effectiveness of the Modified RTK-GNSS to balance the compromise between high availability and high reliability of the fixed positions. On the third test course, the Modified RTK-GNSS had a 6.7% lower fix rate than u-blox F9P but a superior 2DRMS, which suggests that it obtains fewer wrong fixes than u-blox F9P.

The Modified RTK-GNSS was compared against RTKLIB and rtklibexplorer by using the same parameters and optimal parameters. In using the same parameters, Modified RTK-GNSS had the highest fix rate in first and second test courses, with slightly lower by 1% with rtklibexplorer. Nevertheless, it had the smallest 2DRMS, outperforming the two libraries. In using optimal parameters, the Modified RTK-GNSS had a slightly lower fix rate than rtklibexplorer and a higher fix rate than RTKLIB, and it still had the best 2DRMS values among the three. This shows that by using better satellite selection and further validation of the ambiguity solution, a good compromise between the availability and reliability of fixed solutions is achievable. These comparisons also suggest that outputs of RTKLIB and rtklibexplorer depend on the basic parameter setup of the program. This may be an advantage or disadvantage. One user may opt to just set a minimum number of parameters for all of their datasets, while another user may have enough time to test different parameter setups until they obtain the desired output. The opposite is also true for the previous statements. However, our program focuses on developing better algorithms to be used in the RTK-GNSS engine, especially in challenging environments, rather than parameter setup. Regardless of whether the same parameters or optimal parameters are used, Modified RTK-GNSS shows the best performance in most test courses, followed by rtklibexplorer, then RTKLIB. This does not mean that RTKLIB should not be used. Instead, this opens an opportunity for researchers to improve RTKLIB in coping with non-ideal situations of GNSS like urban or dense-canopy environments where multipath errors heavily affect position performance. In fact, rtklibexplorer and Modified RTK-GNSS are good examples. Overall, Modified RTK-GNSS was able to maintain a relatively good percentage of fix rate without degrading its outputs. At the same time, it is also true that not all wrong fixes were removed by our method, as discussed, although it was able to minimize errors relative to the other three RTK-GNSS engines.

## Figures and Tables

**Table 1:** Mask angle and minimum $C/N_{0}$.

Parameter	Value
Mask Angle	10°
Minimum C/N_0_	35 dB-Hz (L1 and L2 bands)

**Table 2:** Threshold based on PDOP.

PDOP Value	Threshold (Cycle)
<1	0.1
1–2	0.2
>2	0.3

**Table 3:** Parameter settings of RTKLIB.

Parameters	Values
Mask Angle	10, 15, 20, 25, 30, 35
Minimum C/No (dBHz)	30, 32, 34, 36, 38, 40, 42, 44
Code/carrier ratio	100, 200

**Table 4:** Parameter settings of rtklibexplorer.

Parameters	Values
Mask Angle	10, 15, 20, 25, 30, 35
Minimum C/No (dBHz)	30, 32, 34, 36, 38, 40, 42, 44
Code/carrier ratio	200, 300

**Table 5:** Horizontal 2DRMS comparisons between Modified RTKLIB and commercial receiver.

Test Number	Modified RTK-GNSS	u-blox F9P
First Test Course	5.12 m	11.88 m
Second Test Course	5.68 m	16.45 m
Third Test Course	8.41 m	7.97 m

**Table 6:** 2DRMS of Modified RTK-GNSS and u-blox F9P.

Test Number	Modified RTK-GNSS	u-blox F9P
First Test Course	0.26 m	0.32 m
Second Test Course	0.96 m	0.82 m
Third Test Course	0.17 m	0.54 m

**Table 7:** Fix rate comparison of Modified RTK-GNSS, RTKLIB, and rtklibexplorer using **the same parameters**.

Test Number	Modified RTK-GNSS	RTKLIB	rtklibexplorer
First Test Course	66.8%	24.0%	44.6%
Second Test Course	60.1%	25.1%	50.7%
Third Test Course	67.5%	41.5%	66.4%

**Table 8:** Fix rate comparison of modified RTK-GNSS, RTKLIB, and rtklibexplorer using **different parameters**.

Test Number	Modified RTK-GNSS	RTKLIB	rtklibexplorer
First Test Course	66.8%	44.1%	64.3%
Second Test Course	60.1%	34.3%	60.8%
Third Test Course	67.5%	54.3%	72.5%

## Data Availability

The raw data supporting the conclusions of this article will be made available by the authors on request.

## Abbreviations

The following abbreviations are used in this manuscript:

$C/N_{0}$	Carrier/noise ratio
DGNSS	Differential Global Navigation Satellite Systems
DD	Double-differenced
ECEF	Earth-centered, earth-fixed
GLONASS	Globalnaya Navigatsionnaya Sputnikovaya Sistema
GNSS	Global Navigation Satellite Systems
GNSS/INS	Global Navigation Satellite Systems/Inertial Navigation Systems
GPS	Global Positioning Systems
ION	Institute of Navigation
LAMBDA	Least-Squares Ambiguity Decorrelation Adjustment
LiDAR	Light Detection and Ranging
LOS	Line of Sight
NLOS	Non-Line of Sight
PDOP	Position Dilution of Precision
PNT	Positioning, Navigation, and Timing
QZSS	Quasi-Zenith Satellite Systems
RMS	Root Mean Square
RTK-GNSS	Real-time Kinematic-Global Navigation Satellite System
TUMSAT	Tokyo University of Marine Science and Technology
2DRMS	Two-dimensional Root Mean Square
